# Practical applications of organizational health literacy: a conceptual framework and implementation in public health

**DOI:** 10.3389/fpubh.2026.1801820

**Published:** 2026-06-08

**Authors:** Alisa Seo-Lee, Melanie Sampson, Simon Kim, Darcie Moeller

**Affiliations:** 1 Cook County Department of Public Health, Forest Park, IL, United States; 2 Literacy Works, Chicago, IL, United States

**Keywords:** health literacy, health equity, organizational culture, organizational change, practical applications

## Abstract

Health literacy is a foundational driver of health equity, yet most efforts have focused on individual skills or clinical communication rather than the organizational systems that shape how people access and use health information and services. Organizational health literacy (OHL) offers a systems-level approach that expands responsibility to include organizations and systems. OHL emphasizes this responsibility between and across individuals, organizations, and broader systems by cultivating systems level focus on designing policies and practices that are easy to navigate and use. This case study describes the development, implementation, and early lessons from a large suburban county health department’s Health Literacy Initiative. Using a practice-informed, user-centered design process grounded in the literature and stakeholder input, we developed an open-access toolkit and complementary learning resources organized around six core domains: leadership and organizational transformation; health-literate workforce development; partnerships and intersectoral collaboration; accessibility of information and services; communication practices and standards; and community engagement and outreach. Although developed for a single geographic jurisdiction, the framework and tools are readily adaptable across diverse public health contexts, offering a practical approach to advancing health equity.

## Introduction

When people consider health literacy in health care and public health, they tend to think about personal health literacy, such as knowing what questions to ask at a doctor’s appointment and being able to read and understand medical information and prescriptions (1). Personal health literacy enables patients to understand their health conditions and fully participate in shared decision-making about their health. It can prevent miscommunication, decrease errors and patient safety events, and improve patient outcomes. People with proficient health literacy skills are better able to navigate complex health systems. In 2020, the U.S. Department of Health and Human Services articulated a two-pronged definition in Healthy People 2030 that differentiated personal health literacy and articulated organizational health literacy. In this framework, organizational health literacy (OHL) was defined as: “the degree to which organizations equitably enable individuals to find, understand, and use information and services to inform health-related decisions and actions for themselves and others” (2).

OHL represents an alternative, systems-based approach to addressing health literacy. It applies principles of universal precautions based on the recognition that public health workers and clinicians cannot reliably identify those people who will have difficulty understanding. OHL has further been discussed as a concept that incorporates cultural competence and humility, responsive practices, patient centered care, patient engagement, and preventive medicine. As OHL “is not only determined by the ability of individuals within a population but also by the responsiveness of health-care systems and services, policy interventions and practice are more likely to be effective if they are not limited to personal intervention but seek to improve the quality of navigation support, information and communication within health-care systems and services” (3). OHL can thus spread population health interventions in a scalable and more cost-effective way than approaches focused on personal health literacy. OHL is also critical in achieving health equity, as it is “about creating the systems and conditions that allow everyone to thrive… improving OHL directly contributes to reducing health disparities…because it ensures that all individuals have the same opportunity to make informed decisions and take control of their health, regardless of the challenges they face… The most effective and equitable approach is not about asking individuals to decode these complex systems. It’s about simplifying the systems themselves” (4).

While it is essential for people to advocate for their own health-related needs, long-standing inequities make that difficult to achieve. The healthcare setting is “laden with structural bias from racialized medicine, a biased learning environment, and poor compositional diversity” (5); bias also occurs as a result of our brains’ attempts to interpret the copious amount of information we take in each day. That said, organizations are responsible for developing policies and practices that “reduc[e] the impact that negative implicit biases have on patient safety and [prevent] adverse patient outcomes” (6). In this context, we describe the development, implementation, and early lessons from a large, suburban county health department Health Literacy Initiative. This case study presents the conceptual framework for the Advancing Organizational Health Literacy toolkit ^1^ (7), describes its development and components, and discusses limitations and future directions.

## Background

Historically, OHL has “been conceptualized as the ability of health care providers to address the information needs of people living with limited health literacy skills” (8). As such, OHL implementation has been situated mostly in the clinical setting, where it is seen as a “communication issue, involving the construction of trusted relations between the providers and the patients” (8). Fortunately, definitions of OHL have changed over time, “reflect[ing] a growing recognition of the need for systemic, equitable, and practical approaches within healthcare organizations to support health literacy. The definitions have progressed from a focus on availability and accessibility to a broader emphasis on equity, systemic efforts, and practical adaptations to enhance health literacy outcomes” (9). Despite this progress, there have been fewer efforts to advance OHL in public health settings, which represents a missed opportunity to leverage these strategies in advancing health equity.

To address this gap, we developed a practice-oriented toolkit and related resources designed to support OHL within public health and social service settings. OHL often operates behind the scenes, shaping how systems are designed, how work is organized, and how organizations prioritize their values. These design choices strongly influence how systems function for communities. In recognition of this principle, we created the toolkit and related resources with an inward lens, enabling organizations to evaluate and align their internal processes with OHL concepts. Our toolkit leveraged OHL facilitators such as “broad participation, engagement, and co-participation” which are “the most frequently reported facilitators” in recent OHL literature (9). Key concepts included the importance of leadership; integration of OHL into ongoing planning, evaluation, and quality improvement; the vital role of the workforce; user centeredness and universal precautions; and the application of clear communication practices internally, down to the interpersonal level (10). We also aimed to support organizations in integrating OHL into their external interactions and relationships to strengthen intersectoral partnerships.

Many interactions related to social determinants of health occur outside of traditional health care settings, including within food pantries, workforce development programs, and other social service and nonprofit organizations. Recognizing that limited foundational understanding of OHL concepts is a key barrier, we extended OHL education to non-healthcare sectors (9). For this work, we defined the public health workforce broadly to include entities across governmental, health care, community-based, and academic sectors, and their employees who provide care, connect people to resources, communicate health information, conduct applied public health research, and/or carry out governmental public health functions.

Given the diversity of settings for which the toolkit was designed, it was intentionally structured to be adaptable across a wide range of public health and social service contexts. Organizations can engage with the toolkit regardless of factors such as setting, size, staffing structures, distribution of responsibilities, available resources, or levels of staff training. This flexibility allows organizations to use the toolkit in ways that are responsive to their specific needs rather than requiring a standardized implementation model.

The toolkit design was carefully grounded in health literacy principles itself - emphasizing accessibility, use of plain language, inclusivity in language and representation, and ease of use. We intentionally developed it to be inviting and engaging—qualities often prioritized in materials created for community members—recognizing that organizational users benefit from the same considerations. These design choices were intended to help organizations see the toolkit as approachable, usable, and applicable to their own work.

As a governmental public health agency that leads and implements initiatives at the county level, we sought to advance OHL within the context of a large and diverse jurisdiction. The county we serve includes more than five million residents and encompasses wide variation in income, race and ethnicity, languages spoken, and access to resources. This context underscored the importance of an OHL approach that could be applied across systems and sectors to support equitable public health practices.

Within this context, our department’s mission, which reflects a commitment to health optimization and the advancement of health equity, informed the development of the Health Literacy Initiative and positioned health literacy as a guiding principle for systems transformation. Our department aims to support “a health literate public health system that enables everyone to achieve their best health by making governmental, health care, and social service systems more navigable for community members of all races, genders, sexualities, socioeconomic situations, and personal health literacy skills” (7).

## Methods

### Health literacy initiative overview

In 2022, using state and federal grant funds, we launched the Health Literacy Initiative. This initiative included funding support for four community partner organizations to engage in OHL work, as well as 12 organizations to support the hiring of community health workers (CHWs) as a strategy to strengthen organizational capacity for health-literate practices. The initiative also supported health literacy training for internal staff and community partners, technical assistance to guide implementation of OHL concepts, and the development of shared tools and resources, including the OHL toolkit, a dedicated website, and related materials. These efforts were complemented by ongoing educational and dissemination activities, including a webinar series focused on advancing OHL in public health.

### Toolkit goals and design principles

The toolkit was designed as a dynamic resource that acknowledged the expertise of the public health workforce, encouraged workers to view their efforts through a health equity lens, and modeled the same health literacy practices promoted within the field. In alignment with this approach, the Health Literacy Initiative had two primary goals: (1) to support the public health workforce in sharing public health information using appropriate languages, modalities, and cultural context, and (2) to increase access to important public health information - including information about social services, programs, and health care - in the virtual and physical environments most used by priority communities (7). We sought to promote the consistent use of health literacy best practices across the local public health system to improve access to social and health care services. To support broad uptake and access, the toolkit was free and publicly available.

### Literature review and environmental scan

Key health department staff identified aspirational goals for the health literacy work, and six areas within which to focus the work. A structured literature review of OHL and existing frameworks followed to investigate focus area themes. Core references in this process were *Ten Attributes of Health Literate Health Care Organizations* by Brach et al. (10) and the CDC attributes of a Health Literate Organization which emphasize organizational responsibility for health literacy rather than framing challenges as individual patient deficits (2). As articulated by Brach et al. (10), “Being a health literate organization is more than initiating a few projects that address health literacy; it means that health literacy is an organizational value… infused throughout the organization and embraced as part of the organization’s core business”. This framing reinforced the principle that OHL must be embedded across organizational functions and grounded in institutional values. Similarly, Abrams et al. (11) highlights the importance of a universal precautions approach, noting that “It is the responsibility of all health team members to use […] clear communications practices with everyone—to make sure all patients, clients, and families understand. This is not an add-on; it is part of everyone’s job.”

We next conducted an environmental scan of publicly available toolkits and initiatives, including resources listed on the Centers for Disease Control and Prevention website. Existing resources and toolkits were targeted towards audiences ranging from community members to healthcare providers. We found an emphasis on clinical settings, a focus on communication skills without sufficient guidance on broader systems change, and limited attention to initiatives within local public health departments. We interpreted these gaps in the context of the real-world experiences of our team and grantee organizations working in the community, and our role in supporting a large, diverse county with communities spanning a wide range of resources, capacities, and needs. These considerations informed the scope and orientation of the toolkit.

### Toolkit development and co-design process

Toolkit development followed an iterative, practice-informed process that incorporated expert input, user experience feedback, and consensus building. Development was conducted in partnership with a local nonprofit organization specializing in plain language. In addition, we collaborated with an academic office of health literacy to deliver training for health department staff and community-based organizations funded through the Initiative. Insights from these trainings and partnerships informed refinement of toolkit content and structure.

### Implementation context and equity considerations

We recognized that organizations within our public health system face persistent challenges, including “complicated communication and information-sharing systems, inconsistent and patchwork funding, and siloed programs and services” (7). Throughout the development process, the project team also reflected on challenges that came up in multiple institutions during program implementation including barriers to staff participation in professional development, communication skills needed to support program delivery, and process challenges encountered by staff working in resource-constrained systems. These challenges cannot be addressed by individual organizations in isolation, underscoring the need to address health literacy on both organizational and systemic levels. A core principle guiding our work was the intentional integration of equity-centered practices within individual organizations as a critical step on the pathway to achieving a health literate public health system.

### Toolkit structure and core domains

Guided by literature, practice experience, and expert consensus, we organized the toolkit around six core domains of OHL: (1) leadership and organizational transformation, (2) health literate workforce development, (3) partnerships and intersectoral collaboration, (4) accessibility of information and services, (5) communication practices and standards, and (6) community engagement and outreach (6). These domains reflect both internal organizational processes and external system interactions central to advancing a health-literate public health system.

### Pilot testing and user feedback

Development of the toolkit was part of a greater initiative which included support for community health workers as a proactive public health intervention across the suburban region. The initiative also included work with six academic, research, and community-based institutions to provide training and technical assistance to grantee organizations. A draft version of the toolkit was pilot tested in spring 2024 with more than 100 individuals through this network including representatives from the community-based organization grantees, health department staff, technical assistance providers supporting grantees, and other partners. This pilot testing included stakeholder interviews, survey feedback, and revision review with our local plain language experts. We utilized a co-design centered approach to include users as collaborators with the goal of creating useful and relevant solutions for our target audience.

Feedback from the pilot testing revealed early use of the toolkit to identify priority OHL areas, with several individuals indicating progress in at least one domain or plans for future implementation. Respondents shared examples of how they used or planned to use the toolkit and related focus areas:

- “[Focus Area] #1 helped us identify [where] we could improve health literacy as an organization.” (Leadership + Organizational Transformation)
- “The cultural humility examples were very helpful and relevant to our ongoing work.” (Communication Practices + Standards)
- “The toolkit helped identify areas of need in the community through surveying the community we serve and how to create goals as an organization for the future.” (Community Engagement + Outreach)

Qualitative feedback was analyzed with an inductive, bottom-up approach. Information was then organized thematically and grouped into strengths and areas for improvement. Strengths related to audience relevance, layout and design, and content accessibility. Users liked its interactive nature, the examples provided, and the engaging infographics. One user noted, “This nuanced approach to health literacy is so wonderful and you have done an amazing job taking a challenging concept and making it accessible.”

Areas for improvement related to document length, navigation, and the need for more background information and structured guidance. The feedback from the survey complemented feedback from interviews and the team’s observations of program needs identified through periodic meetings with grantee representatives, technical assistance with grantees provided by our plain language experts, and learnings from professional development offered to grantees during this initiative.

### Toolkit revision and finalization

Pilot feedback informed revisions including content organization, visual design elements, and development of a companion website to enhance usability and access For example, one suggestion was that “with the six strategies, I think color coding the content could be helpful in navigating the document” The team then integrated a color-coding system that could also be used in the web hub and in supplementary documents to help create cohesion and distinction between sections and themes. In response to suggestions related to document length and navigation, the toolkit was reorganized into clearly defined sections aligned with the six focus areas, with consistent internal structure and visual design elements to support easier navigation.

Feedback also emphasized the importance of practical, actionable content that reflects the realities of working within resource-constrained systems. In response, we strengthened the use of structured guidance, stepwise examples, and visual aids, and more explicitly framed OHL as integral to existing public health work rather than an additional activity. Users also requested greater emphasis on upstream thinking, health equity, and structural determinants of health. One respondent commented, “I would like to see the toolkit go deeper into describing structural determinants of health/structural racism with the inclusion of specific local examples and images. I would also elaborate more on definitions of some key terms that may not be familiar for organizations that do not identify as being part of the public health system.”

**Figure 1:**
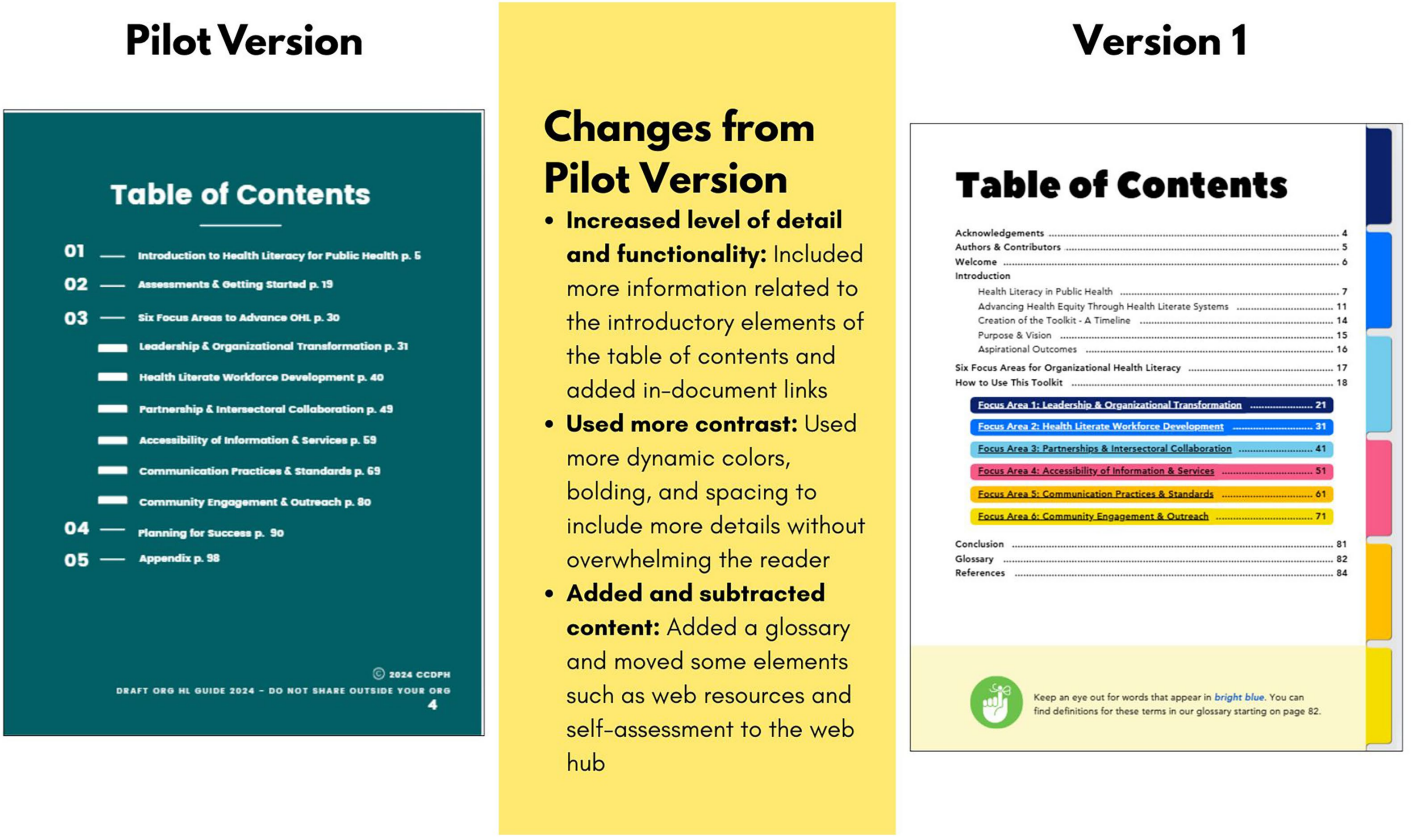
Revisions after pilot version.

**Figure 2:**
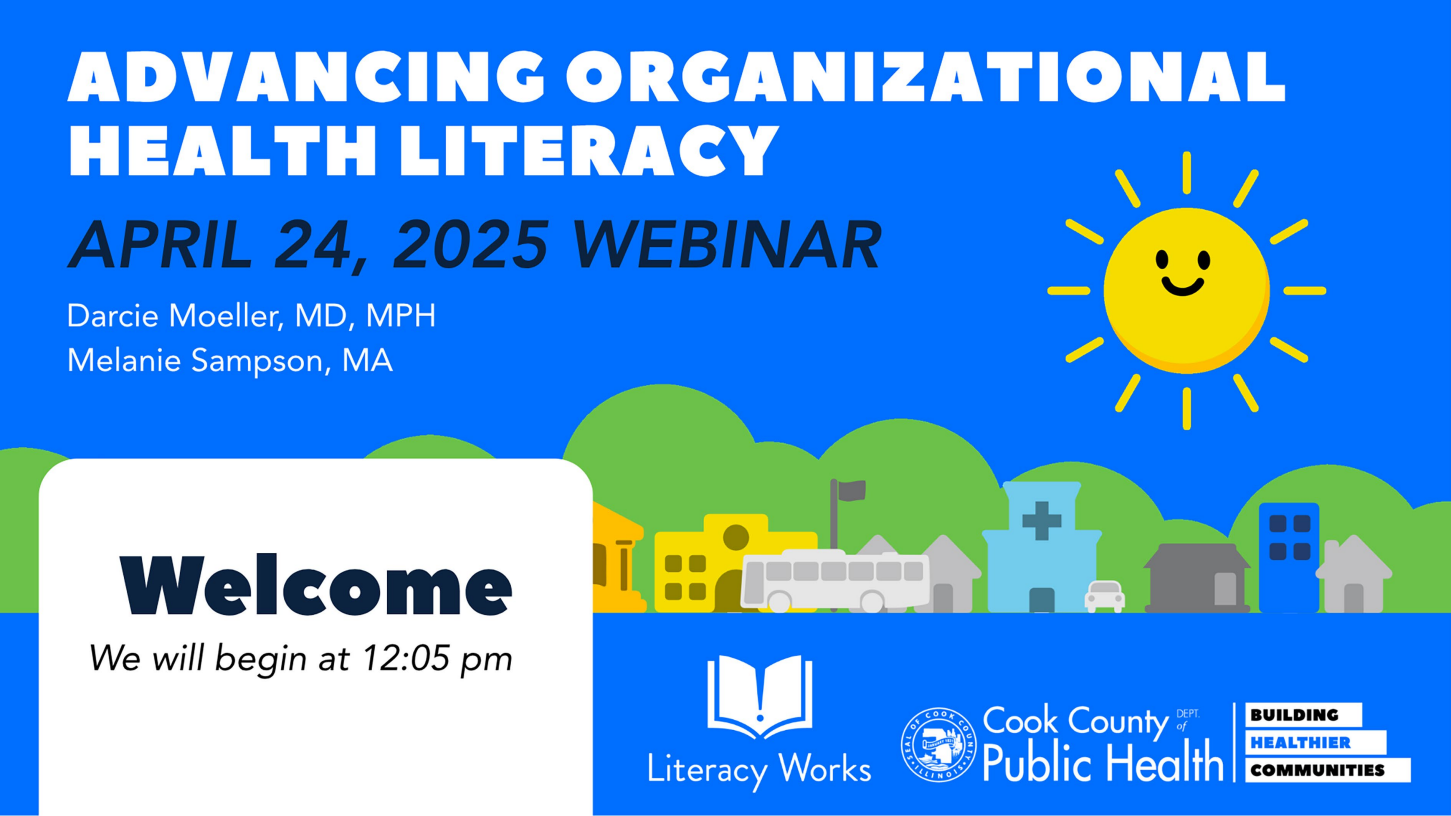
Advancing organizational health literacy webinar.

Accordingly, the revised introduction includes definitions of health equity, an overview of social and structural determinants of health, locally relevant examples, and graphics illustrating the relationship between determinants of health and a health-literate public health system. Additional revisions included expansion of the glossary and references, and development of a companion website to support flexible access and use across organizational contexts. Please see Figure 1 to see examples of the updates made based on feedback. The final toolkit and companion website was launched in the spring of 2025 with an introductory webinar (see Figure 2).

## Results

Our toolkit includes six specific focus areas, which are highlighted by focus area and key objectives, as shown in Table 1.

**Table 1:** Six focus areas for organizational health literacy.

Focus area	Key objectives addressed in the toolkit
Leadership & organizational transformation	Incorporate organizational health literacy (OHL) into goals, values, and work to advance health equity Have OHL built into staff roles and job descriptions Fund and support OHL initiatives and needs, including accessibility, translation, and compensation for lived experience Have OHL-related outcomes assessed and reviewed regularly Have surveys and feedback systems in place to evaluate OHL practices and engage in continuous quality improvement
Health literate workforce development	Attract and retain a diverse workforce by providing living wages, generous paid time off, and other tangible benefits that support a sustainable workforce Deliver effective onboarding and training for new staff Have professional development support and expectations in place for all staff, including leadership and administrators Internally model clear, effective communications and processes Have a culture that supports staff contributions and knowledge sharing
Partnership & intersectoral collaboration	Understand the wide range of local community assets to effectively and holistically serve community members Model organizational health literacy practices when interacting with other organizations Share expertise and knowledge, including effective approaches and lessons learned, with others in the public health system Recognize how power shows up and amplify the voices of communities most harmed by health and racial inequities Offer clear expectations around collaborations and recognize when to take space and when to make space
Accessibility of information & services	Create spaces and programming that are as widely accessible (and Accessible) as possible Design processes with input from community members to ensure they effectively meet their needs Create trauma-informed processes and spaces that promote safety, comfort, and support Have effective internal and external referral systems in place to connect community members to useful resources Have comprehensive translation and interpretation services that all staff know how to access and use effectively
Communication practices & standards	Use a health literacy universal precautions approach in all interactions and communication materials Communicate effectively in plain language, in community-centered ways Adapt messages using a lens of cultural humility, ensuring information is responsive to the needs of the community Make sure staff have a variety of strategies to share information and check for understanding, including in conversations and presentations Communicate effectively through different media to meet community needs, taking care to consider accessibility across formats
Community engagement & outreach	Act as trusted sources Co-design processes, practices, and materials with community members and partners Prioritize community leadership and inclusion in processes and planning Model strong media literacy practices Use data and input from community members’ voices to guide strategies and programming

### Focus area 1: leadership and organizational transformation

This focus area centers on leadership’s role in embedding OHL into organizational goals, values, and routine work to advance health equity. The toolkit emphasizes integrating OHL into staff roles, dedicating resources to support OHL initiatives, and regularly assessing OHL-related outcomes. Feedback mechanisms and continuous quality improvement processes are highlighted as key strategies for sustaining health-literate organizational practice.

### Focus area 2: health literate workforce development

This focus area promotes building and sustaining a workforce equipped to advance OHL. The toolkit showcases strategies to attract and retain a diverse workforce, support effective onboarding and ongoing professional development, and model clear and effective internal communication. It also underscores the importance of organizational cultures that value staff contributions, support knowledge sharing, and recognize the public health workforce as members of the communities they serve.

### Focus area 3: partnership and intersectoral collaboration

This focus area puts a spotlight on the role of building and maintaining strong, cross-sector partnerships and collaborative networks in advancing health literacy. The toolkit focuses on understanding local community assets, modeling OHL practices in organizational relationships, and sharing expertise and lessons learned across sectors. It also places emphasis on the importance of recognizing power dynamics, amplifying the voices of communities most harmed by health and racial inequities, and establishing clear, equitable expectations for collaboration.

### Focus area 4: accessibility of information and services

This focus area features guidance on designing information, services, and environments to be accessible across a wide range of needs and contexts. The toolkit highlights creating accessible spaces and programs, incorporating community input into process design, and applying trauma-informed approaches that promote safety and inclusion. It also reinforces the importance of effective referral systems and comprehensive translation and interpretation services that staff can readily access and use to support community members.

### Focus area 5: communication practices and standards

This focus area emphasizes consistent, clear communication practices as a core component of OHL. The toolkit highlights the use of a universal precautions approach, plain language, and cultural humility across communication materials and interactions. It also raises the importance of checking for understanding, equipping staff with multiple strategies to share information, and ensuring communication across media formats is accessible and responsive to community needs. Strong communication practices and a commitment to self-reflection and improvement are elevated as crucial to create a culture of care (12).

### Focus area 6: community engagement and outreach

Community engagement is presented here as a central driver of OHL, describing how agencies connect with and respond to the populations they serve. The toolkit calls attention to acting as trusted sources of information, prioritizing community leadership and inclusion in planning and decision-making, and co-designing processes, practices, and materials with community members. Our healthcare system and the non-profit sector (sometimes referred to as the nonprofit industrial complex) are knotted, complicated organisms that have friction between community health needs and the demands of the sectors (13). We wanted to ground this section in centering communities while acknowledging this tension.

## Discussion

### Positioning organizational health literacy in public health practice

This case study positions OHL as a systems-level strategy for public health practice, rather than a narrowly defined communication intervention. Our work demonstrates how OHL can be applied to advance health equity and strengthen intersectoral relationships within a large county public health system. Drawing on prior OHL literature, we adapted core principles to reflect the realities of public health organizations and infrastructure. By encouraging organizations to reflect on their own practices and providing concrete guidance for implementation, this approach frames OHL as an organizational responsibility embedded within everyday public health work.

### Organizational health literacy as a strategy for advancing health equity

OHL is a critical strategy for advancing health equity. Prior research demonstrates that “the organizational approach of focusing on the informational needs of patients is an effective strategy to improve patient outcomes and healthcare quality” (14), and that a “social gradient of health literacy” exists, such that health literacy contributes to health disparities (14). We know that inequities are reinforced by how organizations design information, services, and processes. Our work therefore builds on this understanding, emphasizing that organizations can reduce inequities by embedding health literacy principles into everyday operations. By tying OHL directly to health equity, this framing encourages action across public health in working toward this common goal.

### Implications for public health systems

This case study underscores the importance of offering multiple, low-barrier entry points for engagement with OHL. In addition to the toolkit, a complementary webinar series was developed and launched in the fall of 2025. Each webinar aligned to a specific focus area, allowing organizations to engage with content in ways that match their capacity, priorities, and learning preferences. These tools and resources are updated on the relevant focus areas of the website as they are developed, functioning more as a learning community rather than a static product.

Early engagement metrics suggest that this multi-model, open-access approach can support broad reach. Since launch, the main health literacy website has received more than 2,600 page views and has over 1,000 active users. The webinar series brings in more than 60 participants for each webinar, with many more viewing the recording asynchronously. Additional outreach through social media expanded visibility. While engagement metrics do not measure impact, they are critical process measures and suggest that having flexible, freely accessible resources can facilitate participation in OHL professional development.

### Lessons learned

Applying a user-centered design approach reinforced the importance of co-design in developing OHL resources. Incorporating feedback from future toolkit users highlighted the value of predictable structure and consistent organization, particularly for busy practitioners working in complex systems. User input directly informed reorganization of the toolkit into six focus areas with recurring components, making it easy for users to view content section to section. Instead of organizing information by categories such as reflection questions, practical tips, and examples, we used key topics under each of the six focus category areas and used those to drive the bulk of the content with other repeating features such as the local partner spotlight and a snapshot of work within our health department. We repeatedly heard how many directions our partners were pulled in; the toolkit’s organization into six focus areas allowed users to jump into sections which were most applicable and interesting to them, without needing to read the entire toolkit from beginning to end prior to toolkit utilization.

Implementation also reinforced the importance of adaptability and flexibility in public health practice. Practical aspects of the work required ongoing revision in response to shifting circumstances, including changes in funding and operational constraints. For example, the toolkit was initially designed to be available in print, a modality that was abandoned after early-termination of federal funding support.

### Limitations

This case study has several limitations that should be considered. First, the toolkit relies on a web-based format, which may not align with the preferred learning modalities for all organizations. Second, the current version of the toolkit is structured as a unidirectional text, offering limited opportunities for interaction or collaboration with the OHL experts. Finally, this work represents just one approach to advancing OHL, and other models or strategies may be equally effective in promoting health literacy in public health. As a single-jurisdiction case study, findings should be interpreted in light of local contextual factors. However, our work can be more broadly instructional via its focus on meeting the needs of our learning community, which drove multiple iterations of our toolkit to its final product. This work can be replicated with a similar user-centered design process to meet the needs of other communities.

### Future directions

Future work will focus on continued evaluation, adaptation, and sustainability of OHL resources through development of a learning community. Capturing more data on how people are using the toolkit and webinars will support continuous quality improvement. Updating the toolkit to include new or different examples from community partners represents one concrete strategy to maintain relevance over time. Outcomes such as “general OHL improvement” and “facilitated or strengthened responsiveness” are the two most frequently reported in the literature and are important metrics to track in this type of work (9).

Additional areas for future exploration include strategies to sustain OHL activities beyond time-limited funding and adapting or tailoring the toolkit for other settings, such as rural communities. Data on the cost-effectiveness of OHL interventions remain limited and represent an important gap worth exploring, particularly as organizations look for improved efficiency and return on investment. Cost-effectiveness is thus another outcome to study, which is an important shift beyond the evaluation of OHL tools, which is where many studies focus their efforts. Finally, the potential role of artificial intelligence in disseminating, tailoring, and supporting OHL practices warrants further study.

## Conclusion

Organizational health literacy holds an important role in reshaping complex health systems and strengthening public health practice by shifting the burden of navigation and understanding from individuals to the organizations and systems meant to serve them. By designing services, information, and processes to meet the needs of communities, organizational health literacy offers a practical pathway for advancing health equity.

This case study demonstrates how organizational health literacy can be operationalized within a governmental public health setting through a flexible, practice-oriented toolkit and complementary learning resources. Although this work was developed to support a specific county public health jurisdiction, its underlying principles and structure are readily adaptable for use in other public health settings. Throughout this process, we learned many of the same lessons the toolkit seeks to promote, including the importance of user-centered design, adaptability in the face of changing circumstances, and the value of strong intersectoral relationships. By sharing this approach, we aim to support other public health systems in embedding organizational health literacy into everyday practice and working alongside community partners to create more navigable, inclusive, and equitable systems.

## Data Availability

The original contributions presented in the study are included in the article, further inquiries can be directed to the corresponding author.

## Footnotes

1
https://cookcountypublichealth.org/health-literacy-in-public-health/
